# Behavior of Plain Concrete of a High Water-Cement Ratio after Freeze-Thaw Cycles

**DOI:** 10.3390/ma5091698

**Published:** 2012-09-24

**Authors:** Huai-Shuai Shang, Ting-Hua Yi, Yu-Pu Song

**Affiliations:** 1 School of Civil Engineering, Qingdao Technological University, Qingdao 266033, China; E-Mail: shanghuaishuai@yahoo.com.cn; 2 Faculty of Infrastructure Engineering, Dalian University of Technology, Dalian 116023, China; E-Mail: syupu@dlut.edu.cn

**Keywords:** plain concrete, freezing and thawing cycles, mechanical properties, weight loss, dynamic modulus of elasticity

## Abstract

An experimental study of plain concrete specimens of water-cement ratio 0.55, subjected to 0, 15, 25, 40, 50 and 75 cycles of freeze-thaw was completed. The dynamic modulus of elasticity (DME), weight loss, compressive strength, tensile strength, flexural strength, cleavage strength and stress-strain relationships of plain concrete specimens suffering from freeze-thaw cycles were measured. The experimental results showed that the strength decreased as the freeze-thaw cycles were repeated. A concise mathematic formula between DME, weight loss, mechanical properties and number of freeze-thaw cycles was also established. The influences of freeze-thaw cycles on the DME, weight loss and mechanical properties were analyzed. The experimental results serve as a reference for the maintenance, design and life prediction of dams, hydraulic structures, offshore structures, concrete roads and bridges in cold regions.

## 1. Introduction

Since the emergence and first practical use of concrete as a building material in the late nineteenth century, concrete has become one of the most versatile and widely produced materials in the world. Increasing population and living standards in both developing and developed countries will lead to an ever increasing demand for concrete materials. It has been predicted that concrete will continue to be the most popular industrial material [1]. Nevertheless, memorable structures, such as dams of hydroelectric plants, bridges crossing rivers and lighthouses are intended to serve for centuries while being destined to perpetually expose to various attacks by their surroundings.

Concrete has the potential to be damaged if it is subjected to freeze-thaw cycles. The American Concrete Institute (ACI) has established specifications for the protection of concrete placed during cold weather. The ACI defines cold weather as period when more than three successive days have a mean daily air temperature less than 40 F (Fahrenheit). It is thus important to fully understand the mechanical characteristics [2] of plain concrete after freeze-thaw cycles in order to predict the structural response and the structure life. The freezing and thawing durability [3,4,5] of concrete is of the utmost importance in countries with subzero temperature conditions, such as the AA zone, Russia, northern Europe and China, *etc*.

Sun *et al.* [6] investigated damage and damage resistance of high strength concrete under the action of flexural load and freeze-thaw cycles. Bertil Persson [7] outlined laboratory and analytical studies of salt frost scaling and internal frost resistance of self-compacting concrete that contains an increased amount of filler, different air content and dissimilar methods of casting. Cheng *et al.* [8] investigated the influence of freeze-thaw cycles on the compressive strength, flexural strength and cleavage strength of plain concrete. Qin [9] investigated the strength and deformation characteristics of plain concrete under uniaxial and multiaxial compression after different cycles of freeze-thaw, the influence of stress ratio and number of freeze-thaw cycles on the strength and strain were then analyzed. Gokce *et al.* [10] introduced some information about freezing and thawing resistance when air-entrained or non-air-entrained concrete is used as recycled coarse aggregate in air-entrained concrete.

The dynamic modulus of elasticity and weight loss were the main focus for most previous research on plain concrete suffering from freezing and thawing cycles. These studies, however, did not provide information on the strength and deformation of concrete under uniaxial compressive stress states, especially information on the strength and deformation of the concrete specimens under uniaxial tensile stress states. Hence, this paper studied the compressive strength, tensile strength, cleavage strength, flexural strength and the ultrasonic velocity of plain concrete specimens with water-cement ratio 0.55 after different cycles of freeze-thaw according to the *Test Method of Long-Term and Durability on Ordinary Concrete* GB/T 50082-2009 [11].

## 2. Experimental Procedures

### 2.1. Materials and Mix Proportions

In this investigation, local materials were utilized. A Chinese standard (GB175-2007) [12] Type I 425# Portland cement (which has standard compressive strength of 42.5 MPa at the age of 28 days) was used. Natural river sand with fineness modulus of 2.6 was used. Coarse aggregate was a crushed stone with diameters between 5 mm and 10 mm. The mix proportions is as follows: cement (360 kg/m^3^), sand (611 kg/m^3^), Coarse aggregate (1241 kg/m^3^), Water (198 kg/m^3^). The mixing was accomplished after putting all the coarse and fine aggregate into the mixer. These ingredients were mixed for about one minute, then the water was added within one minute. The mixing continued for about two minutes after all water was added. And then the air content (it was 1.9%) was measured. 

### 2.2. Samples and Testing Programs

Concrete prisms with size of 100 mm × 100 mm × 100 mm (to determine the compressive strength, tensile strength and cleavage strength) and 100 mm × 100 mm × 400 mm (to determine the weight, the dynamic modulus of elasticity and flexural strength) were cast in steel molds. The major stress direction was always applied to surface which was perpendicular to the ground surface. All cast specimens (the number of the test specimens is 106) were compacted through external vibration and demolded 24 h later. Thereafter, all the specimens were cured in a condition of 20 ± 3 °C and 95% Relative Humidity (RH) for 23 days. Some of the specimens (the number of the specimens was 85) were then immersed in water for 4 days prior to the freeze-thaw cycles.

The freeze-thaw cycling test was performed following the *Test Method of Long-term and Durability on Ordinary Concrete* GB/T 50082-2009 at the age of 28 days. The temperature of concrete samples was controlled by a Pt sensor embedded in the center of the concrete sample. The temperature of the sample center ranged from −17 ± 2 °C to 8 ± 2 °C. In a single cycle, the temperature of the specimens cools from 6 °C to −15 °C and then warmed to 6 °C all within 2.5–3 h in water. The compressive strength, tensile strength, stress-strain curve, dynamic modulus of elasticity and weight loss of the specimens were tested and recorded at 0, 15, 25, 40, 50 freeze-thaw cycles. At least three specimens were measured for each testing item. Three layers of butter and three layers of plastic membranes were used as a friction-reducing pad when compressive strength was tested.

The tensile tests were conducted in a special triaxial testing machine [13] (designed by State Key Laboratory of Coastal and Offshore Engineering, Dalian University of Technology) that is capable of developing independent compressive or tensile force and can have random ratios. The testing of the specimens can be carried out by strain-control, and the loading speed was 0.002 mm per second. The loads were applied by means of the loading jacks operated by air hydraulic pumps. The jacks were equipped with spherical, self-aligning heads to obtain uniform distribution of stress on the specimens. The tensile strength was measured according to the loading mode in [14]. 

## 3. Results and Discussions

### 3.1. Failure Modes

**Figure 1:**
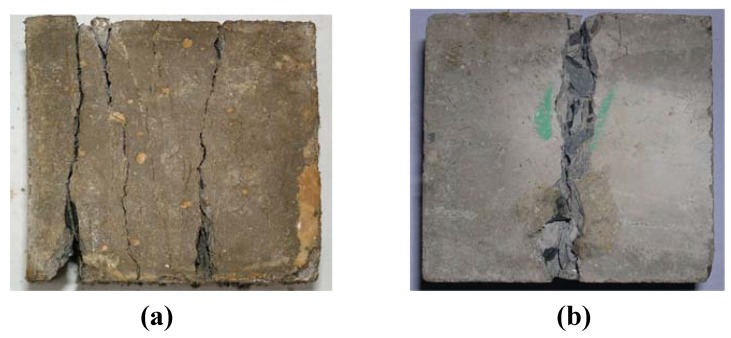
Failure modes of plain concrete prior to freeze-thaw cycles. (**a**) Failure modes under compressive load; (**b**) Failure modes under cleavage load.

Figure 1 shows the failure modes of plain concrete under uniaxial compressive and cleavage load. The splitting tensile strain along the unload plane(s) was the cause of failure for both. It was obvious that the influence of freeze-thaw cycles on plain concrete did not change the splitting tensile mode from occurring according to the experiment. There was no great change in the failure modes for plain concrete specimen after freeze-thaw cycles.

The tensile strain will be caused in the direction of free surface because of the action of compressive load or cleavage load, and the crack forms when the tensile strain was larger than the ultimate tensile strain of the specimen. It was noticed that the cracks on the loaded surface have a random direction because of the influence of coarse aggregates. The failure mode of the specimens under tension load or cleavage load was always instantaneous, controlled by a single crack propagating rapidly at the center of the specimen.

### 3.2. The Relative Dynamic Modulus of Elasticity and Weight Loss

The parts of the relative dynamic modulus of elasticity (RDME) and weight loss of plain concrete of water-cement ratio 0.55 after accelerated freeze-thaw cycling tests are given in Table 1. They were mean values of three specimens.

**Table 1:** The relative dynamic modulus of elasticity (RDME) and weight loss of plain concrete after different cycles of freeze-thaw.

**Numbers freeze-thaw cycles (N)**		**0**	**15**	**25**	**40**	50
RDME /%	Test	100	86.2	74.1	58.8	46.8
	Computed	100.9	84.9	74.2	58.2	47.6
Weight loss /%	Test	0.0	0.40	0.79	1.18	1.78
	Computed	−0.07	0.45	0.79	1.31	1.65

Notes: 25 cycle tested at 31 days, 50 cycles tested at 33 days.

The dynamic modulus of elasticity is the proportion of stress to strain when the stress is least under the action of dynamic loads. The dynamic modulus of elasticity can be measured by means of longitudinal vibration or flexural vibration. It reflects the elasticity performance of material, similarly to the initial tangential modulus under static loads. The loss of the dynamic modulus of elasticity with freeze-thaw cycles means the loss of the elasticity performance. The RDME is the ratio of the dynamic modulus of elasticity measured after a certain number of freeze-thaw cycles to the initial value prior to freeze-thaw cycles. As Table 1 shows: the RDME decreased to 46.8% after 50 cycles of freeze-thaw. Equation (1) is the mathematic expression between the RDME and number of freeze-thaw cycles through analyzed the experimental results according to the least square regression method:

(1) D=100.9338−1.0675⋅N

where *D* is the relative dynamic modulus of elasticity, *N* is the number of freeze-thaw cycles, correlation coefficient is 0.998.

Equation (2) gives the relationship between the weight loss and number of freeze-thaw cycles:

(2) W=−0.0666+0.0344⋅N

where *W* is the weight loss after different cycles of freeze-thaw, correlation coefficient is 0.995.

Table 1 presents the computed results of the RDME and weight loss according the Equations (1) and (2) respectively.

The weight loss of concrete specimens is caused by surface separation or scale off. The rate of freeze-thaw cycling in the laboratory conditions was much higher than in the natural environment. Thus, it is reasonable that the scaling observed was more severe during the freeze-thaw cycles. The reason is as follows: the weight variation of concrete was caused by water movement and surface separation. Concrete is a three-phase composite structure at microscopic scale, a mortar matrix, aggregate and the interfacial transition zone between the two. Microcracks exist at cement paste-aggregate interfaces within concrete even prior to any load and environmental effects. In repeated cycles of freeze-thaw in a wet environment, water will enter the cracks during the thawing process of the freeze-thaw cycle. If the mass of surface separation is larger than the water absorbed by the concrete specimens, the weight of the concrete specimens will decrease.

### 3.3. Strength Characteristics

The tensile stress and the compressive stress were, respectively, calculated by dividing the tensile load and the compressive load by the area of loading section (0.01 m^2^). These values were then used in the analysis of the test results. In this paper, compression is denoted as negative, tension as positive. Table 2 gives the parts of the compressive strength and tensile strength of plain concrete after different cycles of freeze-thaw. They were mean values of three specimens.

**Table 2:** Compressive strength and tensile strength of plain concrete after different cycles of freeze-thaw.

Numbers freeze-thaw cycles (N)		0	25	50	75
Compressive strength /MPa	Test	−19.66	−15.15	−9.95	－
	Computed	−19.78	−14.92	−10.07	－
loss /%		0	22.9	49.4	－
Tensile strength /MPa	Test	1.93	1.36	0.84	0.56
	Computed	1.87	1.40	0.94	0.47
loss /%		0	29.7	56.5	71.2

As seen from Table 2, the tensile strength decreased sharply under the action of freeze-thaw cycles; it gave about a 29.7% decrease over the initial tensile strength after the first 25 cycles of freeze-thaw. Furthermore, after 75 cycles of freeze-thaw, it decreased to about 28.8% of the initial tensile strength. After 50 cycles of freeze-thaw, the compressive strength decreased to about 50.6% of the initial compressive strength.

As the freeze-thaw cycles were repeated, the tensile strength and the compressive strength decreased. The reason is as follows: the microcosmic cracks were caused after the action of freeze-thaw cycles, the direction and distribution of microcosmic cracks are stochastic. The number of the microcosmic cracks increased and the width of the microcosmic cracks become broad as freeze-thaw cycles increased. When the specimen is under the action of compressive loads, the cracks are caused in the direction parallel to the compressive load. When the specimen is under the action of tensile loads, the cracks are caused in the direction vertical to the tensile load. The effective area will become less with the initiation and growth of every new crack. This reduction of the effective area further causes an increase in the stress concentration at critical crack tips.

The results were analyzed by the least square regression method, a mathematic expression between the tensile strength, compressive strength normal value and numbers of freeze-thaw cycles is given out:

(3) $$f_{t}^{D}=1.8684-0.0186\cdot N$$

where $f_{t}^{D}$ is the tensile strength after different cycles of freeze-thaw, correlation coefficient is 0.979.

(4) $$f_{c}^{D}=-19.775+0.1942\cdot N$$

where $f_{c}^{D}$ is the compressive strength after different cycles of freeze-thaw, correlation coefficient is 0.998.

The computed results of the tensile strength and compressive strength according to the Equations (3) and (4) were given in Table 2.

Table 3 gives the flexural strength and cleavage strength of plain concrete after freeze-thaw cycles.

The cleavage strength can be drawn through the Equation as following [15]:

(5) $$f_{ts}=\frac{2F}{\pi A}=0.637\frac{F}{A}$$

where $f_{ts}$ denotes the cleavage strength, *F* is the failure load, *A* is the area under cleavage load.

The flexural strength can be drawn through the Equation as following [15]:

(6) $$f_{ctm}=\frac{Pl}{bh^{2}}$$

where $f_{ctm}$ denotes the flexural strength, *P* is the failure load, *l* is the span of support, *b* is the width of section of concrete specimen, *h* is the height of section of concrete specimen.

As seen from Table 3: the flexural strength and cleavage strength decreased to 54.1% and 55.4% of the initial value after 50 cycles of freeze-thaw respectively. The results were analyzed by the least square regression method, a mathematic expression between the tensile strength, compressive strength normal value and numbers of freeze-thaw cycles is given out:

(7) $$f_{ctm}^{D}=4.5865-0.039\cdot N$$

where $f_{ctm}^{D}$ is the flexural strength after different cycles of freeze-thaw, correlation coefficient is 0.959.

(8) $$f_{tu}^{D}=2.1278-0.020\cdot N$$

where $f_{tu}^{D}$ is the cleavage strength after different cycles of freeze-thaw, correlation coefficient is 0.988.

The computed results of the flexural strength and cleavage strength according the Equations (7) and (8) were given in Table 3.

**Table 3:** Flexural strength, cleavage strength of plain concrete after different cycles of freeze-thaw.

Numbers freeze-thaw cycles (N)		0	15	25	40	50
Flexural strength /MPa	Test	4.49	3.99	3.75	3.20	2.43
	Computed	4.59	4.00	3.61	3.03	2.64
loss /%		0	11.1	16.5	28.7	45.9
Cleavage strength /MPa	Test	2.13	1.85	1.62	1.26	1.18
	Computed	2.13	1.83	1.63	1.33	1.13
Loss /%		0	13.2	23.9	40.9	44.6

### 3.4. Stress-Strain Relationships

**Figure 2:**
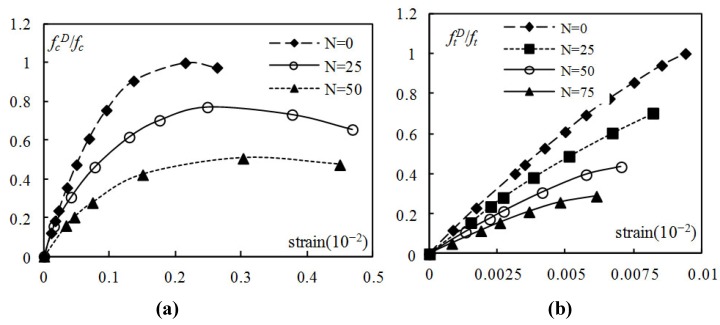
Stress-strain curves under uniaxial compressive (**a**) and tensile (**b**) loading after different cycles of freeze-thaw.

Figure 2 depicts the stress-strain curves of plain concrete specimens of water-cement ratio 0.55 suffering from different cycles of freeze-thaw under uniaxial compressive and tensile load. It can be seen that the stress-strain curves of concrete under uniaxial compression went flat as the freeze-thaw cycles increased, the peak value of strain increased with freeze-thaw cycles increased. While for plain concrete specimens of water-cement ratio 0.55 under uniaxial tension, the peak value of strain decreased with freeze-thaw cycles increased. 

### 3.5. Ultrasonic Velocity

Ultrasonic velocity is the speed at which sound travels through a given material. It is affected by density and elasticity. Velocity remains constant in a given material. After 0, 25, 50, 75, 100 cycles of freeze-thaw, the decreasing percentage of the ultrasonic velocity is 100%, 92.08%, 85.45%, 72.69% and 61.65% respectively. It can be seen the ultrasonic velocity decreased as freeze-thaw cycles were repeated. 

## 4. Discussion

After 50 cycles of freeze-thaw, the RDME decreased to 46.8%, while the compressive strength, tensile strength, flexural strength and cleavage strength decreased to about 50.6%, 43.5%, 54.1% and 55.4% of the initial value, respectively. According to GB/T 50082-2009 and the ASTM C666 Standard Test [16], the specimen reaches failure if the RDME dropped to 60% or less, or its weight loss exceeded 5.0%. However, it can be seen that this criterion for failure of concrete may be not appropriate according to the experimental data. The experimental data within this paper is in agreement with the data in References [8] and [9] (It is not suitable to take the RDME decreased to 60% and weight loss decreased 5% as the damage criterion for plain concrete suffers from freeze-thaw cycles according to the testing result. The experimental results of strength should be considered.). Namely, the loss of strength exceeds 60% of the initial value when the RDME decreased to about 60%.

**Figure 3:**
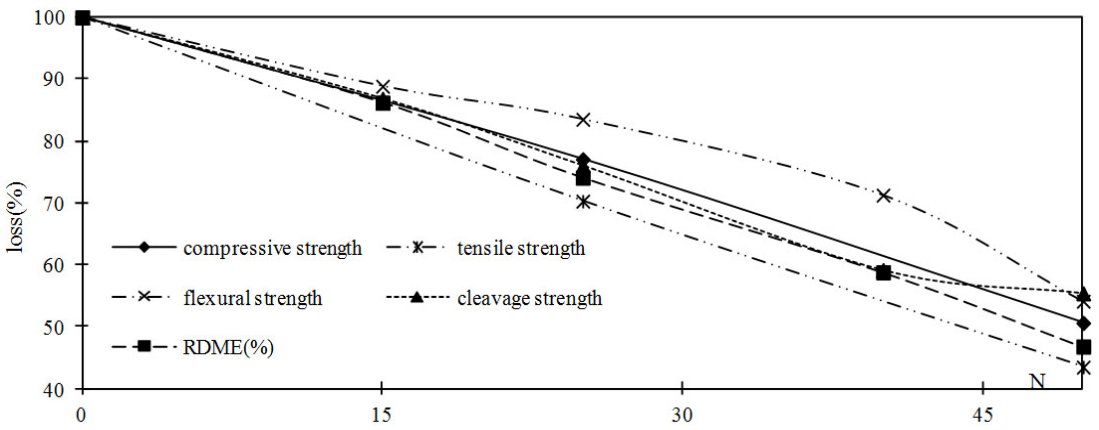
Relationship between change of strength, RDME and number of freeze-thaw cycles.

The effect of freeze-thaw cycles on the dynamic modulus of elasticity, compressive strength, tensile strength, flexural strength and cleavage strength compressive strength is presented in Figure 3.

Concrete is a three-phase composite structure at microscopic scale, a cement matrix, aggregate and the interfacial transition zone between the two. On the other hand, in considering the concrete specimen under the compressive load, tensile load, cleavage load, the initiation and growth of every new crack, the load carrying area will be reduced. This reduction in load carrying area causes a further increase in the stress concentration at critical crack tips. The effective area becomes thus little when the specimen suffers from compressive load, tensile load, cleavage load and the concentration of stress is caused again. The strength decreased as freeze-thaw cycles increased.

## 5. Conclusions

Based on the experimental work in this study and the discussion about the test results, the following conclusions can be drawn:

(1) The compressive strength, tensile strength, flexural strength and cleavage strength decreased as the freeze-thaw cycles increased. The loss of compressive strength and tensile strength after the action of freeze-thaw cycles was evident. According to GB/T 50082-2009 [11], the concrete specimen failed if its RDME dropped to 60% or less, or if its weight loss exceeded 5.0%, but this metewand may be not appropriate according to the experimental data. The strength guidelines should be taken into account.
(2) The stress-strain curves of concrete under uniaxial compression went flat as the freezing and thawing cycles increased. The peak value of strain under uniaxial compression increased with freeze-thaw cycles increased, while the peak value of strain under uniaxial tension decreased with freeze-thaw cycles increased.
(3) The results will help enable structure design and maintenance by considering the freeze-thaw durability of concrete. Therefore, in deciding the damages in concrete caused by freezing, the study of the ice formation process in concrete pores is critical.
